# Human Toddlers’ Attempts to Match Two Simple Behaviors Provide No Evidence for an Inherited, Dedicated Imitation Mechanism

**DOI:** 10.1371/journal.pone.0051326

**Published:** 2012-12-10

**Authors:** Susan S. Jones

**Affiliations:** Department of Psychological & Brain Sciences, Indiana University, Bloomington, Indiana, United States of America; University College London, United Kingdom

## Abstract

Influential theories of imitation have proposed that humans inherit a neural mechanism – an “active intermodal matching “ (AIM) mechanism or a mirror neuron system - that functions from birth to automatically match sensory input from others’ actions to motor programs for performing those same actions, and thus produces imitation. To test these proposals, 160 1- to 2½-year-old toddlers were asked to imitate two simple movements– bending the arm to make an elbow, and moving the bent elbow laterally. Both behaviors were almost certain to be in each child’s repertoire, and the lateral movement was goal-directed (used to hit a plastic cup). Thus, one or both behaviors should have been imitable by toddlers with a functioning AIM or mirror neuron system. Each child saw the two behaviors repeated 18 times, and was encouraged to imitate. Children were also asked to locate their own elbows. Almost no children below age 2 imitated either behavior. Instead, younger children gave clear evidence of a developmental progression, from reproducing only the outcome of the models’ movements (hitting the object), through trying (but failing) to reproduce the model’s arm posture and/or the arm-cup relations they had seen, to accurate imitation of arm bending by age 2 and of both movements by age 2½. Across age levels, almost all children who knew the word ‘elbow’ imitated both behaviors: very few who did not know the word imitated either behavior. The evidence is most consistent with a view of early imitation as the product of a complex system of language, cognitive, social, and motor competencies that develop in infancy. The findings do not rule out a role for an inherited neural mechanism, but they suggest that such a system would not by itself be sufficient to explain imitation at any age.

## Introduction

Imitation is a major route to new knowledge. Imitative learning is generally believed to be especially important for younger organisms, and it has long been established that human infants and toddlers do reproduce the actions of other people [1]–[6]. However, the origins and nature of early imitative behaviors are still areas of active research. By some influential accounts, the ability to imitate the movements of others is innate and evidenced in the behavior of newborn human infants (and newborn rhesus macaque (*Macaca mulatta*) monkeys [5], [7]–[14]). Thus, imitation is important not only as a major social learning mechanism, but also as a possible example of the evolution of a neural mechanism for the inheritance of specific knowledge. Other competing accounts propose that imitation emerges piecemeal during infancy as the product of a complex of processes and has an extended developmental course shaped by interactions between the developing child and his or her environment [3], [6], [15]. Such accounts accord a large role to general processes of learning [6], [15], [16]–[19] and in particular to infants’ learning about their own bodies and movement capabilities and how these map onto the bodies and movement capabilities of others [3], [20]. In the present study, the ability of 1- and 2-year-old children to reproduce two simple modeled movements is measured, and the results are related to these different theoretical accounts of the origins of human imitative abilities.

A central challenge in explaining imitation is solving the “correspondence problem” – that is, explaining how an observer of the overt behaviors of another organism is able to identify the corresponding movements of her own muscles that will reproduce the observed behaviors [16], [21], [22]. At the very least, the imitator must know her own body parts – where they are, how each part moves both alone and in combination with other movements, and how to voluntarily cause those movements to happen. She must also know how all of these map onto conceptually similar but perceptually distinct analogues in the bodies and movements of other people. Accordingly, developmental theories of imitation must explain when and how children develop these kinds of knowledge so that imitation becomes possible.

The most widely accepted account, based primarily on the work of Meltzoff and Moore [4], [11], [12], [23], holds that much of this knowledge is inbuilt, in the form of an innate mechanism that automatically matches seen behaviors to motor programs for the performance of those same movements. Meltzoff and Moore have reported a number of studies in which newborn infants matched simple behaviors modeled by an adult experimenter, and the researchers have interpreted that matching as imitation [4] (but see [1], [15], [24], [25] for different interpretations of the same data). Meltzoff has suggested that the ability to imitate is “…part of a basic human biological endowment” and that “… [newborn] infants can at some level apprehend the isomorphism between body transformations they see and those they feel themselves make” [26]. An *Active Intermodal Mapping* (AIM) mechanism is the proposed mechanism by which newborn infants are able to imitate the movements they observe [12], [23]. In the hypothesized AIM mechanism, visual input specifying an observed arrangement of body parts in another person leads automatically to the activation of the infant’s stored representation of movements that will produce the same arrangement of body parts, leading in turn to the production – the imitation – of those movements. Thus, newborns are expected to imitate only those movements that are already in their repertoires. These would include movements that they have repeatedly performed *in utero*. Prenatal practice of such movements could build or organize or strengthen the “motor programs” that constitute the performance half of the proposed mechanism. However, the AIM proposal does not explain the genesis of the other half – that is, how visual input from others’ behavior might come to be linked to such motor programs by the time the infant is born. Infants have no prenatal visual experience of others’ behavior. Thus, the proposal that the AIM mechanism is operative at birth appears to entail the claim that newborns inherit the knowledge needed to perceive the configurations of body parts produced by others, and then to make a cross-modal link between those perceptions and the sequences of their own movements that will produce the same configurations.

A second influential account of the nature and origins of imitation proposes that human imitation is at least in part the product of a *Mirror Neuron System* (MNS). Such a system is a candidate for the mechanism that achieves intermodal matching of observed and executed behaviors in the AIM model. Mirror neurons are neurons that are active both when a particular action is sensed, and also when that same action is performed. Such neurons were discovered through single cell recordings in adult rhesus monkey cortex [27], [28]. Neuroimaging during imitation by human adults finds activation in cortical areas analogous to the areas of monkey cortex where mirror neurons are found. These areas in human cortex are thought to also contain mirror neurons [21], [29], [30], although the evidence is largely indirect and still controversial [31], [32]. The proposed ‘sensory-motor’ character of mirror neurons suggests that such cells might make human imitation possible by directly linking sensory input from observed actions to motor programs for those same actions [28], [29], [33].

Although the human MNS has been studied mostly in adults, some writings have speculated on its ontogenetic origins [9], [10], [29], [30] and in light of reports of imitation in newborns, suggested that the MNS is already functioning at birth, and explains the newborn infant reported ability to match the behaviors of self and others. The suggestion that mirror neurons explain newborn imitation necessarily implies that some mirror neurons encode or access inherited knowledge of body parts and their movements and of how these map between different bodies – because again, newborn infants have not had opportunities to observe the behaviors of others and so cannot have learned to link such observations to representations of their own movements. A mirror neuron system therefore cannot be a mechanism for newborn infants’ matching of their own and others’ behavior unless mirror neurons are innately preprogrammed. Perhaps for this reason, Meltzoff and Decety [34] have recently expressed reservations about equating mirror neurons with the AIM mechanism, stating that “… it must be underscored that newborn humans are different from both monkeys (who exhibit mirror neurons but little imitation), and from human adults. More analytic work is needed to determine whether the current convergences between the AIM hypothesis (on the psychological level), mirror neurons, and shared representations (on the neuroscience level), and other aspects of social understanding (at the philosophical level) are mere surface similarities or more substantive.”

In an earlier paper, Meltzoff [35] suggested the possibility that mirror neurons acquire their responsiveness through postnatal experience. Other researchers have pursued postnatal learning accounts – in particular, the suggestion that mirror neurons acquire their combined sensory and motor responsiveness through Hebbian learning during self-observation of self-generated action [17], [19], [21], [33], [36]–[38]. By the Hebbian learning account, the repeated simultaneous activation of sensory and premotor neurons – for example, as infants watch their own hand movements – potentiates existing synaptic connections between them [33], so that such cells will subsequently be activated whenever the infant either performs or observes those same movements. Similar paired activations very close in time could occur when caretakers imitated infants’ behaviors, as they very frequently do [18], [20], [25], [39].

The AIM model and the two MNS accounts of imitation are similar in that each focuses on a single, rather low-level automatic process at the core of an imitation mechanism. The AIM account and the inherited MNS account, by relying on evidence of newborn imitation, suggest that this automatic process is a more or less complete mechanism for imitation. The idea that mirror neurons might be created through Hebbian learning could represent a proposal for a complete imitation mechanism, but it is also compatible with a systems view [40]–[42] of imitation. By this view, the ability to match others’ movements develops in infancy as the emergent product of a complex of different kinds of social, cognitive, and motor knowledge, skills and motivations, all with different developmental origins involving the interplay of gene actions, environmental inputs, and the other developing components of the system [40]–[42]. A population of mirror neurons with activations acquired from experience could constitute one component of such a system.

The systems account is consistent with evidence that the most commonly reported instances of newborn infants’ behavioral matching in imitation studies are probably not imitation, and thus that imitation is not innate [3], [26], [43], [44]; evidence that toddlers younger than 30 months have little accessible knowledge about their own or others’ bodies [44]; evidence that preschoolers’ imitation relies heavily on cognitive processing [45]–[47] and especially, evidence that imitation emerges, not all at once as a unit, but at different ages, for the same behaviors in different children, and for different behaviors in the same children [25], [48]–[50].

The question that motivated the present study was whether very young children behave as though they have inherited an AIM and/or MNS imitation mechanism that is functional from birth; whether they behave as though they have an MNS imitation mechanism with mirror neurons that acquire specific activations given sufficient experience; or whether they behave as though their ability to imitate depends on a range of different kinds of acquired knowledge, and so varies widely both between and within different age levels [25].

**Figure 1:**
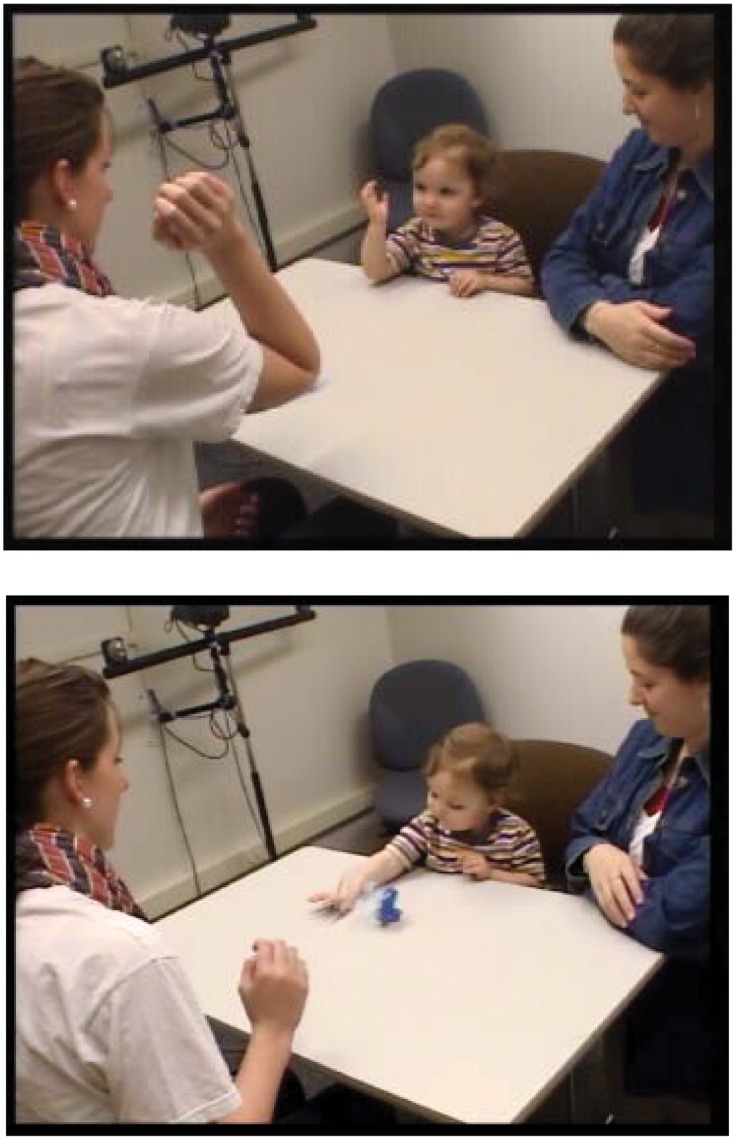
Examples of accurate and inaccurate imitation. Top Panel: unprompted imitation of forearm-raising by a 22-month-old girl as the model raised her forearm for the first time. (This instance of imitation is a particularly accurate match to the model’s behavior– more accurate than the coding criteria required). Bottom Panel: The same child then hit the plastic cup, not with the bent elbow as the model had, but with the side of her wrist.

Two experiments are reported. In Experiment 1, 1- and 2-year-old children were tested for imitation of each of 2 simple behaviors modeled as a single sequence. The first behavior was raising a forearm to bend an elbow (Figure 1, top). Raising the forearm was chosen because it is for toddlers both easy and frequent, and is in these ways like the one response most commonly and convincingly matched by newborns – that is, tongue protruding [1]. By far the largest body of evidence for newborn imitation consists of newborn infants sticking out their tongues at higher than baseline rates in response to an adult model doing the same. Forearm-raising, like tongue protruding, is a simple, directional action. Newborns outside of imitation experiments produce tongue protrusions at high frequencies in response to a range of arousing visual, auditory, and tactile stimuli [3]. Similarly, infants and toddlers bend their elbows by raising their forearms at high frequencies in their everyday lives – for example, each time they bring Cheerios or other objects to their mouths. Thus, if neonates are able to match simple, directional actions like tongue protrusions via an inherited AIM mechanism or MNS, those same infants at 1 and 2 years of age should be able to match forearm-raising using the same mechanism. Moreover – and this is an advantage over tongue protrusion – every sighted toddler has had the opportunity to observe innumerable instances of her own forearm-raising. It follows that, if infants have mirror neurons that acquire their activation profiles after birth through Hebbian learning, both 1- and 2-year-old toddlers should have mirror neurons programmed to be active during forearm-raising, and should be able to match that movement. Thus, the AIM and MNS proposals appear to predict that most children at all age levels in the present study should respond to a model who bends her forearm upward by bending their own arm in the same way. In contrast, a systems account does not predict such uniform findings, because whether or not individual toddlers match the model’s bending of her elbow will depend upon the developmental status of the different system components – for example, whether a particular child has acquired the requisite knowledge about his own and other people’s elbows [3], [45], [47], [50]. By a systems account, then, variability both within and between age levels in how children respond in the imitation task is expected.

The second behavior modeled for toddlers in Experiment 1 was separated from the first by a brief pause (approximately 3 s), during which the bent arm posture was maintained by the model. The second behavior was moving the bent elbow laterally to hit a plastic, spouted cup (a “sippy” cup familiar to most toddlers) lying on a table. Moving a bent elbow side to side (e.g., to reposition an object during oral exploration) is another simple behavior common enough to be in most toddlers’ repertoires. The addition of this behavior to elbow-bending served two purposes. First, using the bent elbow to hit the cup made the modeled 2-action sequence a goal-directed behavior. Mirror neurons in macaque cortex are activated only during goal-directed actions [51]. Secondly, although moving the elbow from side to side was likely to be a well-practiced behavior for most toddlers, using the side-to-side action of the elbow to hit a drinking cup was almost certain to be a novel action with a low probability of spontaneous production. Thus, if children bent their elbows and then hit the cup with the bent elbow, both actions could be more confidently characterized as imitation.

A total of 160 children between 14 and 31 months of age watched as both actions were modeled 9 times by the experimenter and another 9 times by the parent. Children could imitate the bending of the model’s arm at any time during the 18 demonstrations. After each set of 3 demonstrations, the child was given the cup and encouraged to imitate the modeled behaviors, so had 6 opportunities to imitate hitting the cup with a bent elbow. At the end of the experiment, 113 of the children were asked to show the experimenter their elbow.

Experiment 2 was designed to establish whether toddlers would imitate a model’s forearm-raising action if it was presented alone, without the possibly distracting sequel of hitting the cup. Experiment 2 also determined whether children’s forearm-raising behaviors coded as imitation in Experiment 1 were in fact the same behaviors as the well-practiced action that children use to bring finger foods to their mouths.

Twenty-eight toddlers not involved in Experiment 1 (10 from 13 to 15 months of age; 18 from 20 to 23 months of age) completed the same 6-trial protocol used in the previous experiment, except that in each trial, the experimenter or parent model just raised his or her forearm 3 times to make an elbow. There was no cup present. On the first trial, the model said nothing, to see if the child would spontaneously imitate. During each of the remaining trials, the experimenter and parent encouraged the child to imitate (e.g, “Your turn. You do it!”). Following the 6 imitation trials, each child was given a quantity of Cheerios cereal at table level and videotaped while eating this finger food. The time spent eating, the pace of eating, and the number of Cheerios eaten varied widely among children depending on such factors as whether the child had recently eaten, how much they wanted to leave the table, and how much and for how long the parent wanted the child to eat. Because of this variability we report, not the mean number of times children met our criteria for the same forearm-raising action that had been modeled, but rather the number of children who performed that forearm-raising action and the proportion of children’s instances of transporting food to the mouth that were that action.

## Results

### Experiment 1

Video recordings of the experiment were coded for the 6 behaviors listed in Table 1. Behaviors 2 to 5 are actions on the cup ordered by increasing similarity to the model’s demonstrations. Coders noted if infant behaviors followed a parent’s verbal prompt that included the word “elbow”.

**Table 1:** All 9 behavioral measures, with percent agreement and Cohen’s kappa for two judges coding the records of the same 40 subjects.

Behavior	% Agreement	Cohen’s Kappa
1. Imitated fore-arm raising:		
a. Prompted with “elbow”	100	1.0
b. No prompt with “elbow”	95	.90
2. Held/played with cup	95	.77
3. Hit cup conventionally	73*	.21
4. Hit cup unconventionally	82.5	.63
5. Hit cup with bent elbow:		
a. Prompted with “elbow”	97.5	.79
b. No prompt with “elbow”	97.5	.93
6. Named or showed elbow:		
a. Spontaneously	97.5	.66
b. When asked	97.5	.91

* See text.

**Figure 2:**
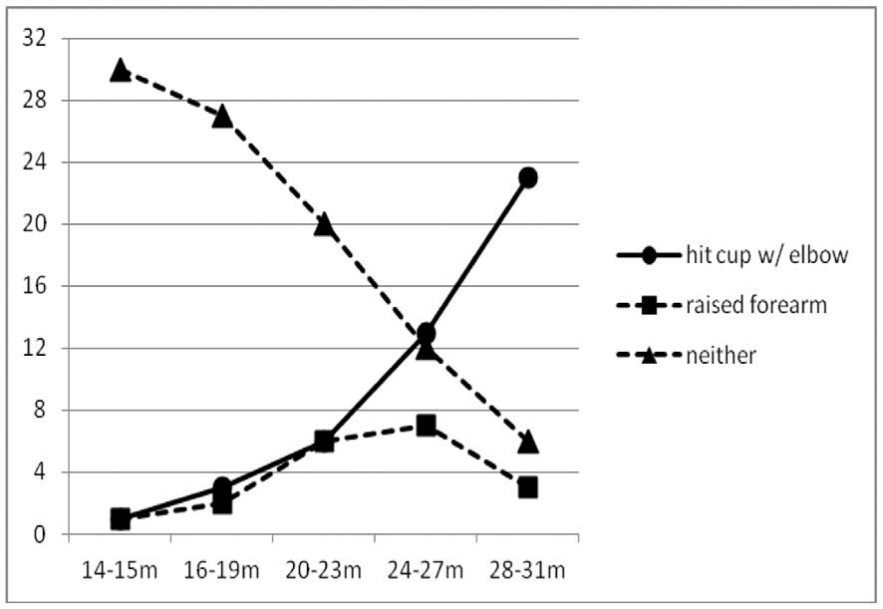
Most toddlers below the age of 2 failed to imitate 2 simple behaviors already in their repertoires. Numbers of children out of 32 in each of 5 age groups who imitated only 1 of 2 modeled behaviors – raising a forearm by bending the elbow (“raised forearm”); imitated 2 of 2 modeled behaviors – bending the elbow and then using it to hit a plastic cup (“hit cup with elbow”); or imitated neither behavior (“neither”).

Figure 2 shows the numbers of children at each age level who raised their forearms and used the bent elbow to hit the cup; raised their forearms but did not hit the cup with the elbow; or produced neither action. Only 65 of the 160 children (40.6%) raised their forearms. As shown in Figure 2, these children were distributed very unevenly across the different age groups (*X* ^2^(4) = 17.48, *p*<.002; Cramer’s V = 0.37): it was not until children were well into their third year that a majority in any age group matched this simple action even once during the experiment.

Forty-six (71%) of the 65 children who raised their forearms after seeing either model do so also used the bent elbow to hit the spouted cup. Thus, children who reproduced the model’s first action were highly likely to also reproduce her second action. Only 8 (17%) of the 46 children who hit the cup with their elbows were verbally prompted by parents using the word “elbow”.

To determine whether children were more likely to imitate the parent’s side-by-side action than the experimenter’s spatially translated action, we noted the trials in which children first bent an elbow without hitting the cup, and the trials in which children first bent an elbow and also hit the cup. Thirteen of the 46 children who did hit the cup had raised a forearm to make a bent elbow during at least one trial prior to the trial in which they first used the elbow on the cup, Nineteen of the 65 children who bent their elbows never did subsequently hit the cup. Thus, there were a total of 32 first trials in which individual children performed only the forearm raising action, and there were 46 first trials in which children raised their forearm and used the resulting bent elbow to hit the cup. Twenty-one of the 32 first instances of forearm-raising (65.6%: compared with chance = 50%, *X* ^2^_(1)_ = 1.03, *p* = 0.31, Cramer’s V = 0.16), and 28 of the 46 first instances of hitting the cup with the bent elbow (61%: compared with chance = 50%, *X* ^2^_(1)_ = 0.70, *p* = 0.40, Cramer’s V = 0.11) occurred during the first block of trials, when the experimenter was the model and sat across the table from the child. Thus, there was no general advantage either of parental or side-by-side modeling or of experimenter or face-to-face modeling in eliciting infants’ imitation.

Of the 19 children who bent their elbows but never did use the bent elbow to hit the cup, 3 children (in different age groups) raised their forearms and simultaneously hit the cup with the hand of the other arm: 15 raised their forearm then lowered it to hit the cup with their wrist or forearm (Figure 1); and 1 25-month-old boy hit the cup with his hand and then bent his elbow.

**Figure 3:**
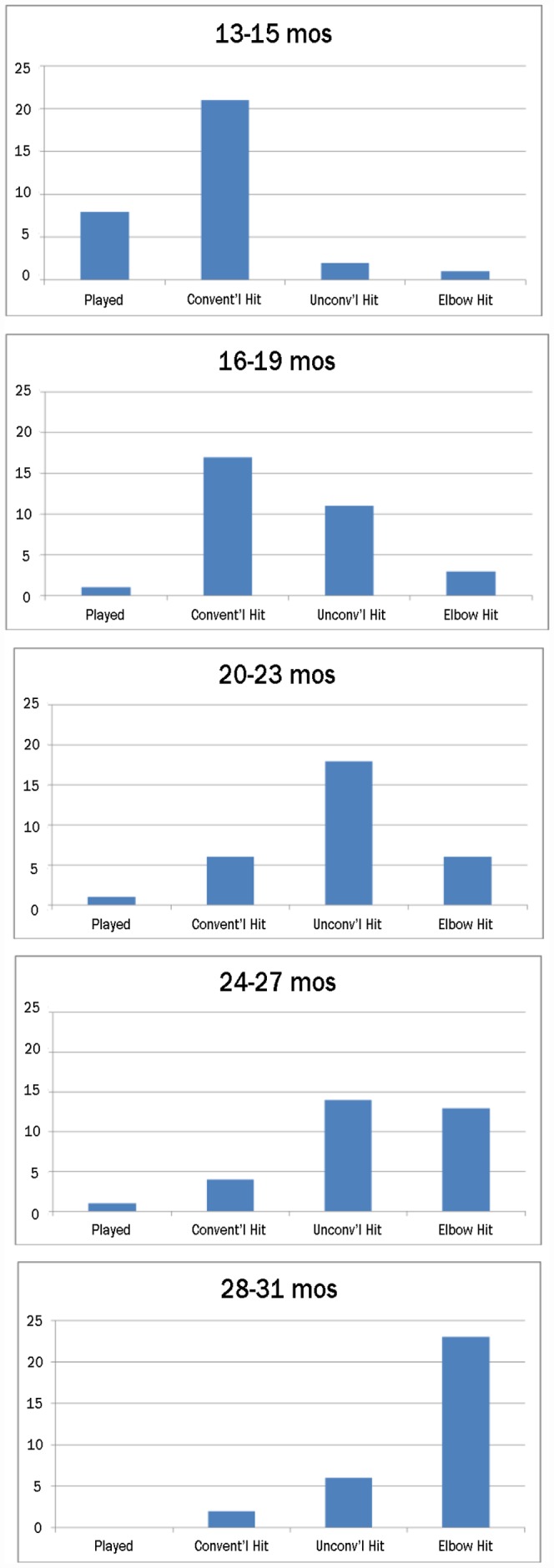
Imitation develops. Children’s closest matches to a model’s multiple demonstrations of hitting a spouted cup with her elbow indicate an extended developmental course for imitation of that action. Four categories of behavior typify the developmental course from the lowest to the highest of the 5 age levels tested (n = 32 at each level): in young 1-year olds, 1) play with no apparent attempt to imitate (“played”), and 2) reproduction, not of the model’s movements, but of the effect of those movements (“conventional hitting”); in most children from 1 ½ to 2 years of age, 3) inaccurate attempts to produce the model’s arm postures and movements (“unconventional hitting”); and in most children by 2 ½ years of age, 4) accurate reproduction of the modeled arm posture and movement (“elbow hit”).

The 5 coded categories of actions on the cup are listed in Table 1 in order of their increasing resemblance to the model’s behavior. Only 2 of the 160 children (1 20–23 month old; 1 29–31 month old) failed to act on the cup in any way. The 158 children who did act on the cup were each categorized by their highest level response in any trial. Figure 3 shows the frequencies of children in each of the 4 categories of actions on the cup at each age level. The figure as a whole shows a clear and protracted developmental course for imitation of this movement. The illustrated distributions of the 32 children at each age level across the 4 behavioral categories differed significantly between all pairings across ordered age levels (Chi-squared tests: *X^2^*_(3)_ ranging from 8.39, *p*<.04, to 20.56, *p*<.0001; Cramer’s V ranging from 0.36 to 0.45) except in the comparison of 20–23-month-olds with 24–27-month-olds (*X^2^*_(3) = _0.29, *p* = 0.29, Cramer’s V = 0.24).

None of the youngest children hit the cup with a bent elbow. About 2/3 hit the cup with the palm of an open hand. Thus, these children were acting to produce the same outcome as the model had produced – that is, the cup’s movement – but gave no evidence that they were attempting to match, or had even perceived, either of the model’s two specific arm movements.

**Figure 4:**
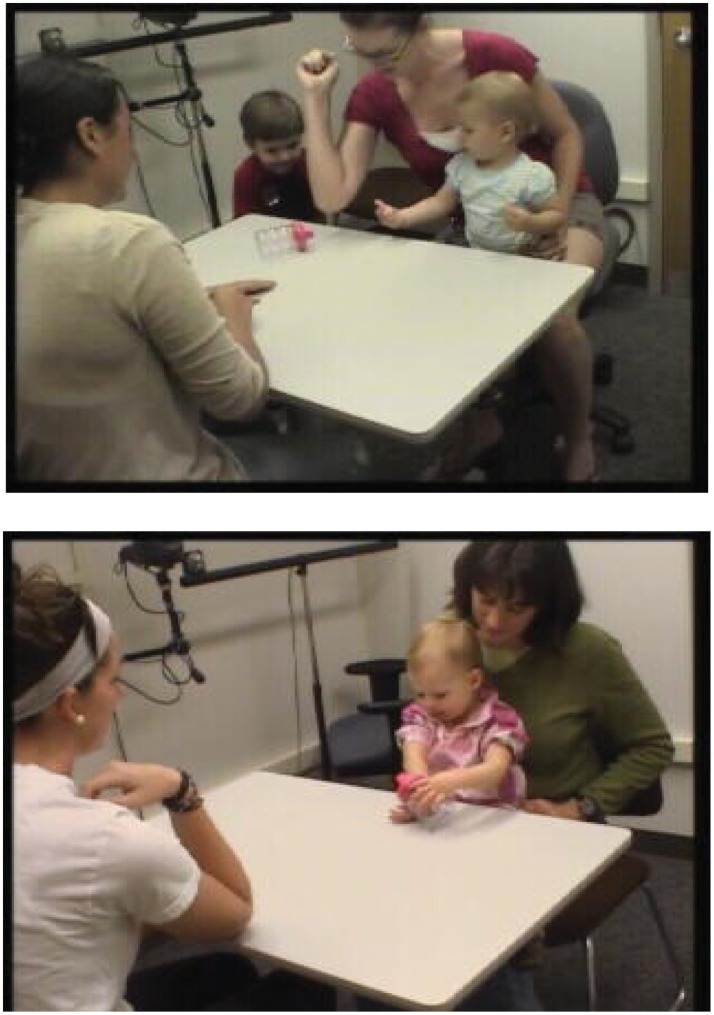
Children’s attempts to visually match a modeled behavior. Top Panel: After watching her mother hit a plastic cup with her elbow, a 14-month-old girl visually compares her mother’s bent elbow to her own bent wrist. Bottom Panel: After seeing the experimenter hit the cup with her elbow, a 24-month-old girl positions the cup on various parts of her own arm, as though attempting to reproduce in vision the modeled relation between cup and arm.

With increasing age, children were less likely to hit the cup “conventionally”, with an open hand, and more likely to hit it “unconventionally” – with a backhanded swiping motion, with a wrist or forearm (Figure 1), or with odd, twisting, or circling motions of the hand or forearm. A small number of children looked back and forth between the model’s arm and their own while trying (but failing) to reproduce the model’s arm posture (Figure 4, above); others tried different ways of positioning the cup next to their arm (Figure 4, below). Such children appeared to be trying to reproduce the visual appearance of the model’s action. Children who actually imitated the model’s forearm-raising were small minorities until 23 months of age. Then, at 2 years of age, more than 62% of children, and at 2 ½, more than 80% of children readily raised their forearms: 41% and 72% of all children in those two groups went on to use their elbows to hit the cup.

If, as seems likely, many instances of unconventional hitting were failed attempts to copy the model, then attempted imitation (unconventional hits+elbow hits) was observed in a maximum of 9% of 13–15 month-olds, 44% of 16–19 month-olds, 75% of 20–23 month-olds, 84% of 24–27 month-olds, and 91% of 28–31 month-olds. Thus, by this measure, attempts to copy the model’s movements – not just to reproduce the outcome of those movements – were not typical of toddlers until the second half of the second year. Being *able* to imitate lagged attempting to imitate by a further 5 to 11 months (Figure 4): it was not until 28–31 months that a majority of children successfully imitated either raising the forearm or using the elbow to hit the cup.

The period from about 18 to 24 months is characterized by rapid word learning. The word ‘elbow’ is known by only a minority of children until sometime after 30 months of age [50]: however, there was a strong link between imitation and knowledge of the word “elbow” in the present study. Table 2 shows how many of the 113 children asked to locate their elbows did so, and how many of those had imitated the model’s arm-bending action during the preceding experiment. Children who understood the word ‘elbow’ and could locate their own elbows were dramatically more likely to have previously raised their forearms in imitation of the model than children who did not understand that word (*X* ^2^(1) = 62.0, *p*<.0001, Cramer’s V = 0.76).

**Table 2:** The relation between having a concept of elbow (showing or pointing to an elbow when asked, or spontaneously producing the word “elbow”) and bending an arm to make an elbow in imitation of a model.

Making an Elbow					
	Showed/Said Elbow		Did Not Show/Say Elbow		
Age Group	Imitated	Did Not Imitate	Imitated	Did Not Imitate	Total
13–15 mos	1	0	1	12	14
16–19 mos	0	0	3	23	26
20–23 mos	5	1	2	17	25
24–27 mos	13	0	3	11	27
28–31 mos	14	1	2	4	21
Total	33	2	11	67	113

It was possible that children’s imitating was linked to their locating their elbows by the 3^rd^ variable of sociability: that is, that outgoing children did both and shy children did neither. We indirectly measured sociability by counting the number of utterances produced during the experiment by each child, except those in the youngest group, who produced almost no utterances, and 2 additional children whose audio records were not complete. There remained 97 children who had been asked to show their elbows –42 who had imitated and 55 who had not. The number of utterances ranged from 1 to 21 in children who imitated (*M = *5.93, *SD* = 5.35) and from 1 to 29 in children who did not (*M = *7.38, *SD* = 7.80), and there was no difference between the group means (*t* (95) = 1.04, *p* = 0.30). Thus, it appears that the link illustrated in Table 2 was not a general one between speaking to the experimenter and imitating the experimenter, but specifically between knowing the referent of the word “elbow” and imitating the bending of the elbow.

### Experiment 2

Because most children below the age of 2 years failed to imitate either of the modeled behaviors, Experiment 2 was designed to test whether (1) children younger than 2 typically could perform the forearm-raising action as it was measured in Experiment 1; and (2) whether younger children would notice and imitate the forearm-raising action if not distracted by the prospect of hitting the cup. Results from children aged 13–15 months were most relevant to the question of whether infants are born with the ability to imitate simple actions that are already in their repertoires. Two of the 10 children in this age group raised a forearm to make an elbow, compared with 2 of the 32 children in the same age range in Experiment 1 (see Figure 2: Fisher’s Exact Probability = 0.24). One child refused to eat any Cheerios, but all 9 of the other children brought cereal to their mouths between 1 and 6 times, and in 100% of these instances, this action met the criteria for forearm-raising used in coding data from Experiment 1. This is hardly surprising, given that the forearm-raising behavior was chosen for modeling because it was thought to be the typical action that young children use to bring finger foods to their mouths. These results reinforced the conclusion from Experiment 1 that children this age failed to imitate the model because they could not imitate, not because they could not easily perform the measured action.

Children from 20 to 23 months were included in Experiment 2 because this was the oldest age level included in Experiment 1 in which imitation was not typical. Results from the children aged 20–23 months were most relevant to the question of whether children’s imitative abilities were underestimated in Experiment 1 because children excited about hitting the cup failed to attend to the model’s specific arm movements. Nine of the 18 children in this group raised a forearm to make an elbow, compared with 12 of 32 children in the same age range in Experiment 1 (*X* ^2^_(1)_ = 0.31, *p* = 0.58, Cramer’s V = 0.12). The frequency of imitation in this age group was not significantly greater than that in the 13–15-month-olds (Fisher’s Exact Probability, one-tailed = 0.124), but there was clearly a trend towards more children imitating as age increased.

Again, one child in this group did not eat Cheerios, but each of the other 17 children brought one or several Cheerios to their mouths from 1 to 6 times, and as in the younger children, 100% of the actions by which they brought the Cheerios to their mouths met the criteria for forearm-raising used in coding data from Experiment 1. Thus, children between 1 and 2 years of age repeatedly produced the forearm-raising action while eating but were no more likely to imitate that action when it was modeled in isolation than when it was paired with the second action of using the bent elbow to hit a cup.

## Discussion

The present experiments are the first direct test in very young children of four current accounts of the origins of imitation in humans (or perhaps three accounts, if the AIM and innately programmed MNS hypotheses are combined). The procedures were designed to optimize conditions for toddlers’ imitation of modeled behaviors via an innate active intermodal matching mechanism (AIM), and/or an innate mirror neuron system (MNS), or a system of mirror neurons that had acquired their activation patterns postnatally, through Hebbian learning during self observation of self movement. By the 4^th^ account - that is, a systems view of imitation as the emergent product of a range of motor, cognitive, and social components - imitation of these actions would not be expected until individual children had acquired the requisite knowledge in each of these broad domains.

In Experiment 1, children from about 1 to 2½ years of age were tested for imitation of each of two simple movements – bending the arm to make an elbow, and then moving the bent elbow sideways to hit a plastic cup. These movements were chosen because they appear to fit theoretical criteria for behaviors that children should imitate via 3 of those 4 proposed imitation mechanisms. In the event, most of the children did not imitate the modeled behaviors. Imitation of one or both of the model’s specific movements was confined almost entirely to children around 2 years of age or older. In addition, imitation was very strongly predicted by whether or not children knew the name and/or location of their elbows.

The finding that children below 2 years of age rarely imitated the movements modeled in this study conflicts with existing reports of imitation of some specific behaviors at much younger ages [11], [12], [26], [53]–[56]. A detailed deconstruction of this research is beyond the scope of the present article, but Jones [3] has argued that many instances of infant and toddler behavior which have been interpreted as imitation are more likely to be something else (for discussion of a range of alternative interpretations of matching behaviors, see [57]). For example, the bulk of the observed newborn behavior that has been interpreted as imitation is tongue protruding – but this is a behavior that newborns spontaneously produce when aroused by any of a range of stimuli [3], [24], [44] suggesting that the sight of a tongue-protruding adult is just another arousing stimulus that happens to match the newborn arousal response it elicits.

The present experiments address theoretical accounts of infants’ reproduction of observed movements, not of observed outcomes. In many reports of imitation by infants late in their first or early in their second year [52], [54], [56], the infants reproduce, not the specific movements made by a model, but the effects of those movements on objects. It seems likely in many cases that the infants have learned about features or affordances of the objects by watching the model’s actions, then acted to exploit those features or affordances rather than to imitate the model [58]. When infants do match the model’s specific behaviors, they seem to have little alternative and so may be acting independently and not imitating [52], [59]. For example, infants in Barr et al. [52] saw an adult pull a mitten off of a puppet’s hand, then pulled the mitten off themselves. Clearly, the infants learned from watching the model that the puppet’s mitten could be removed. What is not clear is whether the infants’ pulling action was imitation of the model’s movement, or just the only way to get the mitten off. The fact that infants did not imitate the two additional behaviors in the three-action sequence modeled for them supports the latter explanation. Similarly, in a study by Elsner and Aschersleben [59], infants learned from observation of a model’s behavior that each of two actions – pressing down or pulling on a ring – produced a different outcome. However, infants in a control group who never saw the two actions modeled produced both actions just as often as the infants who did see the model’s demonstrations. Thus, there is no evidence that infants in the experimental groups were imitating the model’s movements, as opposed to doing what infants in any case would do with the ring. Studies that have focused on infants’ imitation of specific movements have found first imitations of a number of different movements distributed across the second year, and more common in the second half of that year than in the first [25], [48]–[50], [60], [61] In some studies, a small number of infants appear to imitate the model’s behavior early in the second year, but such imitation does not become typical of an age group until several months later in that year [25], [60], [61]. Thus, there is ample precedent for the present results.

For some readers, the imitation task in the present study may bring to mind the tasks used in recent research on rational imitation and the analysis of the rationality of others’ actions by human infants [62]–[65] and by non-human primates [66], [67], and raise questions about how the present results compare with the results of those studies. In the first experiment on rational imitation in human infants [62], 14-month-old infants appeared to behave as though they had analyzed a model’s action on an object in terms of its relative efficiency for achieving the apparent goal of the action, while also taking into account situational constraints on the model’s behavioral choices. According to the researchers, when the model’s choice of an inefficient behavior appeared to be dictated by her circumstances, infants did not imitate the inefficient behavior but produced the more efficient action; whereas, when the model’s choice of an inefficient behavior appeared to be free and unconstrained, infants imitated the inefficient action, as if they had concluded that the model must have had some good reason for choosing that action.

The present study presents what might appear at first to be parallels to the work on rational imitation, in that infants could have reasoned that hitting a cup with a hand is more efficient than hitting it with an elbow and that models who hit the cup inefficiently using an elbow must have had good reason to do so and therefore should be imitated. However, there is no evidence in this study that infants produced such inferences and there is some indirect evidence that they did not. In the present study, infants the same age as Gergely et al.’s subjects [62] and older than those in both Schweier et al. [63] and Zmyj et al. [64] watched the model use her elbow to hit the cup when this action was unconstrained - that is, when her hands were free. But unlike infants in studies reporting rational imitation, these infants did not imitate the inefficient action. Instead, they hit the cup with their hands.

Older infants did try to imitate the unconstrained model’s inefficient action, but had trouble reproducing that action (which is, of course, the major finding in the study). It might be argued that these infants did carry out a rational analysis of the model’s action and decided it was freely chosen, and that this analysis accounts for their attempts to imitate her. However, this argument can not be made because there was no comparison condition in which the model’s inefficient action (hitting the cup with her elbow) was dictated by some environmental constraint, and in which infants did not attempt to imitate that constrained inefficient action. In the absence of such a condition, the present study does not address the question of whether human infants (or adult non-human primates) carry out rational analyses of the actions of others and are guided in their own actions by those analyses.

What, then, do the present results have to say about the different theories of the origins and nature of the mechanism for human imitation? If children in the present study had only failed to imitate the two modeled movements and done nothing else, those negative results might be explained away. For example, children’s failure to imitate might be explained as a consequence of inhibition of the production component of the imitation mechanism: after all, no-one imitates everything she sees. However, the children in this study who did not imitate *did* do something else; and for all but the youngest children, that something appeared to be an active but unsuccessful attempt to imitate.

As already stated, most of the youngest children focused on the effect of the model’s action – the displacement of the cup. They copied this by hitting the cup with their open hands or fists. This behavior demonstrated that the children were happy to engage in the task and take turns with the experimenter and parent. But there was no evidence that the youngest children noticed or responded in any way to the specific movements of the models.

In contrast to the youngest children, toddlers in the middle of the age range did not just hit the cup: many used unconventional arm parts and arm and hand postures to do so. These odd movements suggest that these children *did* notice the model’s movements, noticed that there was something unusual about them, and attempted to reproduce that unusual way of hitting the cup. The fact that children younger than 2 generally did not imitate the model’s initial arm-bending and therefore did not succeed in copying what the model actually did to the cup suggests either that they had not accurately perceived the model’s movements, or that they could not *voluntarily* produce a motor match. In either case, it seems clear that most children in the middle of the age range did focus on the model’s movements and that many of them tried to imitate those movements but received no help either from an AIM (with an innately programmed mirror neuron system or any other innate matching mechanism) or a mirror neuron mechanism with learned activations.

Children who placed the cup close to different arm locations, or looked back and forth between the model’s bent arm and their own while adopting different arm postures (Figure 4) appeared to be trying to reproduce the visual display created by the model’s action – that is, to produce a copy by acting *on,* not directly *with*, the relevant parts of their own bodies. This behavior strengthens the inference that the visual input from the model’s movement was not internally linked to a motor program for the same movement. In short, there is no evidence in this study that children inherit an AIM mechanism and/or a mirror neuron system prepared from birth to produce imitation by automatically linking observed behaviors to motor programs for the same behaviors.

Similarly, there is no evidence from this study to suggest that children were developing a mirror system with activations acquired through simultaneous observation and performance of specific movements [36]–[38], [68]. This lack of evidence does not show that the mechanism does not exist – only that such a mechanism did not appear to produce imitation of a well-practiced behavior in toddlers. Prior studies using neuroimaging have reported experimentally-acquired activations in adults. It has been shown, for example, that professional adult dancers watching others dance show more activity in premotor cortex (as well as in other areas of the mirror neuron system) when observing movements that they themselves have been trained to do than when observing movements that are not in their repertoires [60]. Catmur and her colleagues [36], [37] actually trained erroneous activation patterns in adults by the repeated pairing of the observation of one movement with the production of another. Thus, activation patterns in areas of the mirror neuron system can clearly be learned. But dancers [68] did not dance when they observed their own steps danced by others. Obviously, acquired links between observation and motor activation do not lead to obligatory imitation. However, we know little about how factors other than observation contribute to the initiation and shaping of an observer’s attempt to reproduce an observed movement. The present data indicate that one important factor, even in very young children, might be cognitions about the nature and purpose of the movements in question. The lack of such cognitions might help to explain why so many of the younger children in this study, all of whom had bent their elbows countless times, did not imitate that movement when they saw it. Adults focused on learning new movements are engaged in meaningful behavior. They are likely to attend closely both to the sight of the model’s movements and to the sight and ‘feel’ of their own performance. However, very young children may not attend to and thus may not be conscious of their own forearm-raising when it occurs in the service of eating or oral exploration. Adults also commonly rely on named concepts to mentally represent, organize, understand and compare their own and others’ body parts and movements. That such cognitive processing may be a necessary component of imitation is suggested by the present findings that almost all of the children who knew the word “elbow” and could locate their own elbows were able to imitate the model, whereas children who did not give evidence of knowing the word also did not imitate. It appears that imitation of the model’s actions was mediated by conceptual knowledge about elbows, accessed via the word. The fact that 8 of the 46 children who hit the cup with an elbow did so only after hearing the parent say “elbow” suggests the same thing. The word may have recruited children’s knowledge of their own and other elbows to help them attend to, make sense of, and remember what the adult was doing, so that they could subsequently bend and use their own elbows in the same way.

It has been suggested that cognitive processing is added to direct behavioral matching as imitative abilities develop [46]. The present results indicate instead that cognition may be central to movement matching from the first. Cognitive processing in imitation may not always be conscious. Adults, for example, often unconsciously mimic others’ movements: however, even unconscious mimicry reflects in-the-moment cognitive processing of such factors as interpersonal similarity, ethnicity and relative social status [69]. In very young children, the foremost important cognitive component of an imitation system may be conceptual knowledge of body parts and their movements: and verbal labels (like the word “elbow”) may be an important, even necessary tool for creating, storing and accessing that knowledge.

In this context, it seems relevant that the rhesus monkeys in which mirror neurons were first identified were also engaged in meaningful behaviors – that their mirror neurons were active only when the behaviors observed or performed were goal-directed. It is necessarily true that conscious recognition or voluntary production of any action involves activation of a concept of that action, which of course involves activation of some set of neurons. Thus, Gallese and colleagues [70], [71] have developed the idea that mirror neurons, even in monkeys, might function primarily as components of cognitive appraisal and categorization of the actions of self and other.

In summary: Toddlers in this study did not behave as though they possessed an innate mechanism for imitation, or as though they possessed a mirror neuron system with acquired activations that was sufficient in this age group for the voluntary imitation of specific movements observed in others. Instead, the children provided evidence of a protracted developmental course for imitation of the movements tested, even though those movements were already in their repertoires. Imitation when it did occur was strongly associated with children’s possession of accessible knowledge of the name and location of the body part involved. Together, the findings suggest that the ability to imitate specific movements is not inherited as a purpose-built neural system, but emerges piecemeal, largely in the second year of infancy, and depends upon the development of a range of contributing components including general-purpose mechanisms of learning, language, attention, and cognition.

## Methods

### Ethics Statement

This research protocol, and specifically the use of children as research subjects, was reviewed and approved by the Institutional Review Board at Indiana University. The procedure was discussed with each child’s parent and written informed consent was obtained before the experiment began. Parents of each child portrayed in the figures have also given separate written informed consent, as outlined in the PLoS consent form, to publication of their own and their child’s or children’s photograph(s).

### Participants

A total of 160 children aged 13 to 31 months participated in Experiment 1. Participants were divided into 5 age groups (13–15 mos, *M* = 14.46, *SD* = 0.43; 16–19 mos, *M* = 17.34, *SD* = 1.4; 20–23 mos, *M* = 20.75, *SD* = 1.28; 24–27 mos, *M* = 25.31, *SD* = 1.3; and 28–31 mos, *M* = 29.84, *SD* = 1.0) with 32 children (16 males) in each group. Eight additional subjects did not complete the experiment (6), or experienced procedural errors (2). Twenty-eight additional children participated in Experiment 2. Ten (5 males) were aged 13 to 15 months (*M* = 14.46, *SD* = 0.43), and 18 (8 males) were aged 20 to 23 months (*M* = 14.46, *SD* = 0.43). Participants were identified from county birth records and recruited by letter and telephone. All were full term births with no diagnosed physical or developmental disorders. A large majority were White and middle class, reflecting community demographics.

### Procedures

For both experiments, the procedure began in a laboratory play area where toddler and experimenter engaged in floor play with toys while the full procedure was explained to the parent and written informed consent was obtained. With rapport established, the experimenter, parent, and child moved to a small testing room. Experimenter and parent sat on opposite long sides of a 92 cm×76 cm table. The child sat either on the parent’s lap or in a chair beside the parent.

In Experiment 1, the experimenter first presented an empty spouted cup to the child (“Look at this!”) to handle and examine. No child bent an elbow or hit the cup during this period. When the child stopped handling the cup or after 20 s, the experimenter retrieved the cup, placed it on the table, and said “Look what I can do.” She next raised her right hand vertically to bend her elbow, paused for 2–3 s with her elbow bent and hand roughly at jaw height, then tapped the cup with her elbow, causing it to twirl or slide across the table. This action was repeated 2 more times. The experimenter spoke only to ensure that the child saw both actions during each demonstration (e.g., “Watch me!”). She then placed the cup within 15 cm and at the midline of the child’s torso and said “Your turn. You do it!” Thus, matching of the movement and/or the outcome was explicitly encouraged. The child was given 20 s in which to raise a forearm and/or act on the cup. This sequence was repeated 3 times. Note that children could imitate forearm-raising during any of the 18 demonstrations (see Figure 1) as well as during any of the 6 response periods.

Next, the parent repeated the entire 3-trial sequence. The parent-as-model trials were included to maximize imitation if it was the case that children would more readily imitate the caregiver than a novel person, or would more readily imitate a side-by-side action that required no spatial translation than a face-to-face action that did require spatial translation. However, because children in this age group resist separation from their caregivers, these two variables were entirely confounded with each other, Both were also confounded with the order of the 3-trial blocks.

Together, Experimenter and Parent trials provided each child with 18 models of the two actions, 18 opportunities to imitate bending the elbow, and 6 opportunities to imitate hitting the cup with the elbow. The procedure was digitally recorded for later manipulation checks and coding of the children’s actions.

The word ‘elbow’ is not typically produced before 30 months of age [52], and experimenters did not say “elbow” during testing. However, some early subjects spontaneously named or showed their elbows, prompting us to ask the remaining 113 subjects “Where’s your elbow? Show me your elbow!” at the end of the experiment.

In Experiment 2, the procedure was the same except that no cup was ever present and only the forearm raising action was modeled, 3 times in each of 3 trials by the experimenter, and then 3 times in each of another 3 trials by the parent.

### Coding

For Experiment 1, the 6 behaviors listed in Table 1 were coded from the video records by one judge with limited knowledge of the purpose of the experiment. Children’s forearm-raising was coded as imitation if it met 3 criteria: 1) the movement began during or after the experimenter’s first demonstration; 2) the angle formed by the child’s forearm and upper arm exceeded 90° when the movement began, and was less than 90° when it ended; and 3) the movement raised the forearm vertically such that the angle between the forearm and the table exceeded 45°. Figure 1 (top) shows an ideal example. Note that most instances of forearm-raising that satisfied the coding criteria for imitation were less perfect replicas of the model’s forearm-raising than the instance illustrated here. Coders also noted whether each instance of elbow-bending after the model’s demonstration was or was not preceded by a parent’s verbal prompt using the word “elbow”.

Two of the 160 children did not touch the spouted cup at all. Four categories captured the rest of the children’s actions on the cup: 1) played with or held the cup; 2) hit the cup conventionally, with an open hand or fist; 3) hit the cup unconventionally, with the back of the hand, with the hand or arm in an odd posture, or with the wrist or forearm; 4) hit the cup with the bent elbow. For the 4^th^ category, coders also noted whether or not the child’s action was preceded by parental prompting with the word “elbow”. All behaviors involving the cup were coded according to the above scheme. Finally, the judge coded whether or not children named or showed (pointed to, clasped) their elbows either spontaneously or when asked at the end of the experiment.

A second judge coded 40 (25%) children’s behaviors in 3 sets of 9, 14, and 17 subjects. Levels of agreement between the two coders for each behavior across all 40 records are shown in Table 1. Cohen’s kappas on 8 of the behaviors indicated high levels of agreement. On the 9^th^ behavior, “hit the cup conventionally”, the judges agreed on only 72.5% of instances (kappa = 0.21, “fair” agreement). Disagreements on the first 2 sets of subjects’ data were resolved by joint viewing and discussion. In the final 17 records, agreement on independent judgments of conventional hits was 88%.

For Experiment 2, only forearm-raising was coded. The same 3 criteria used to identify this behavior in Experiment 1 were again used in this experiment to identify the same forearm-raising action produced both during the experiment itself and during the child’s eating of finger food. Two judges independently coded all 28 subjects. There was no inter-coder disagreement about whether any subjects produced behavior meeting the criteria while eating. There was inter-coder disagreement on whether 3 subjects, 1 in the younger group and 2 in the older group, bent their arms to criterion during the modeling of forearm-raising. All 3 children were entered into the analyses as instances of imitation.
